# A mathematical approach to virus therapy of glioblastomas

**DOI:** 10.1186/s13062-015-0100-7

**Published:** 2016-01-07

**Authors:** Victor Lopez de Rioja, Neus Isern, Joaquim Fort

**Affiliations:** ICREA/Complex Systems Laboratory, Departament de Física, Universitat de Girona, Girona, 17071 Catalonia Spain

**Keywords:** Biophysics, Oncolytic virus, VSV, Glioblastoma, Front propagation speed, Reaction-diffusion equations

## Abstract

**Background:**

It is widely believed that the treatment of glioblastomas (GBM) could benefit from oncolytic virus therapy. Clinical research has shown that Vesicular Stomatitis Virus (VSV) has strong oncolytic properties. In addition, mathematical models of virus treatment of tumors have been developed in recent years. Some experiments in vitro and in vivo have been done and shown promising results, but have been never compared quantitatively with mathematical models. We use in vitro data of this virus applied to glioblastoma.

**Results:**

We describe three increasingly realistic mathematical models for the VSV-GBM in vitro experiment with progressive incorporation of time-delay effects. For the virus dynamics, we obtain results consistent with the in vitro experimental speed data only when applying the more complex and comprehensive model, with time-delay effects both in the reactive and diffusive terms. The tumor speed is given by the minimum of a very simple function that nonetheless yields results within the experimental measured range.

**Conclusions:**

We have improved a previous model with new ideas and carefully incorporated concepts from experimental results. We have shown that the delay time *τ* is the crucial parameter in this kind of models. We have demonstrated that our new model can satisfactorily predict the front speed for the lytic action of oncolytic VSV on glioblastoma observed in vitro. We provide a basis that can be applied in the near future to realistically simulate in vivo virus treatments of several cancers.

**Reviewers:**

This article was reviewed by Yang Kuang and Georg Luebeck. For the full reviews, please go to the Reviewers’ comments section.

## Background

Since early last century, viruses have been studied as experimental agents for cancer treatment. The medical interest in the field has fluctuated during this period, reaching a fever pitch in the past two decades. It was in the early 1990s, when recombinant DNA technology became standard, that virus engineering could provide scientific furtherance to virotherapy. Then, oncolytic viruses appeared to be a treatment of tremendous potential and scientists started manipulating them to target cancerous cells more specifically. This culminated in the first marketing approval of an oncolytic virus, granted by the Chinese government in November of 2005 [1]. Very recently, improvements in patient survival have led to endorsements of other oncolytic virus in Europe and the US [2]. In parallel, mathematical models of virus treatment of tumors have been developed [3–5]. However, even with this new ability to engineer viral genomes, a realistic therapeutic frontrunner has yet to emerge.

### Experimental background

Among a variety of aggressive and deadly brain tumors we could highlight the glioblastoma. GBM is the most common and malignant brain cancer. Usually, treatment relies on chemotherapy, radiation and surgery. However these treatments are ineffective and the median survival time of a patient is no longer than 15 months (4 to 5 months without health care), due to multifocality of the disease, infiltrative growth and substantial tumor genotypic variability, among other factors [6, 7]. So, nowadays there are no known medical or surgical approaches that constitute an effective treatment of GBM, and for this reason it is widely considered that the treatment of GBM is likely to benefit from oncolytic virus therapy.

Oncolytic viruses—including retroviruses, herpesviruses and adenoviruses—are an emerging therapy tool for cancers that currently lack effective treatment [8]. The efficiency of different viruses against various tumor cell lines have been studied with in vitro and in vivo experiments [9, 10]. Of these, vesicular stomatitis virus (VSV) has been shown in laboratory studies to have excellent capabilities to become one of the most valuable candidates for virotherapy, due to its very fast lytic cycle and its rapid oncolytic action. In addition, VSV is an enveloped, negative-strand RNA rhabdovirus that can infect a wide variety of species including mice and humans, though it is usually asymptomatic for human beings [11]. Therefore, the anticancer activity of mouse models can be transferable to human trials [12]. This fact makes VSV a strong oncolytic candidate and it has been used in preclinical studies of numerous cancer types, like glioblastomas.

Hence, we focus our attention on the development of a mathematical model of the VSV-GBM virus-tumor system. In the absence of in vivo data, all of the parameter values that we will introduce in the model are extracted from in vitro VSV-GBM experiments. Our main objective is to develop a simple model that can reproduce the VSV-GBM dynamics and explain satisfactorily the experimental in vitro propagation speeds.

### Previous mathematical approaches

The most basic mathematical model of the competition between populations was constructed by Alfred J. Lotka and Vito Volterra in 1925 and 1926 independently [13]. For years their model was improved and adapted to different parasite-host systems, including virus infections [14–17]. Nevertheless, we are interested in a specific model which studies the dynamics of an oncolytic virus through a tumor cell population.

In Ref. [5], Wodarz et al. noted that the few previous reaction-diffusion models of oncolytic virus spread [18, 19] include, in addition to basic spatial dynamics, one or more additional assumptions that introduce further complexity. In contrast, they opt for a very simple approach to the infection process with spatial dynamics. The process of adsorption of a virus *V* by a susceptible tumoral cell *T* (with rate *k*_1_), and replication of *Y* viruses that leave each infected cell *I* (with rate *k*_2_), is essentially described by the reactions

(1) $$ V+T\overset{k_{1}}{\longrightarrow}I\overset{k_{2}}{\longrightarrow}Y\cdot V.  $$

Wodarz et al. study the behavior of an in vitro adenovirus in human embryonic kidney epithelial cells, experimentally and computationally, developing a simple model with two equations (see Eqs. (5) and (6) below), one for susceptible tumoral cells and one for infected cells. They make use of partial differential equations (PDEs) to model the virus-tumor system, because PDEs provide efficient information on the spatial and reactive mechanisms affecting the wave propagating fronts and PDEs can be used to compute their speeds.

The model by Wodarz et al. [5] is a two-equation system that was derived from a three-equation model due to Nowak and May [20]. Including diffusion and logistic growth, the Nowak-May model is

(2) $$ \begin{aligned} \frac{\partial\lbrack \!V](r,t)}{\partial t}&= D_{V}\frac{\partial^{2}[\!V](r,t)}{\partial r^{2}}+k_{2}Y[I](r,t)\\ &\quad - k_{3}[\!V](r,t), \end{aligned}  $$

(3) $$ \begin{aligned} \frac{\partial\lbrack \!T](r,t)}{\partial t} & =D_{T}\frac{\partial^{2}[\!T](r,t)}{\partial r^{2}}\\ &\quad +a[\!T](r,t)\left\{ 1-\frac{[\!I](r,t)+[\!T](r,t)}{k}\right\}\\ &\quad -k_{1}[\!V](r,t)[\!T](r,t), \end{aligned}  $$

(4) $$ \begin{aligned} \frac{\partial\lbrack \!I](r,t)}{\partial t} & =D_{I}\frac{\partial^{2}[\!I](r,t)}{\partial r^{2}}-k_{2}[\!I](r,t)\\ &\quad +k_{1}[\!V](r,t)[\!T](r,t), \end{aligned}  $$

where [ *T*], [*I*] and [ *V*] are the concentrations of susceptible tumoral cells, infected tumoral cells and viruses, respectively; *D*_*T*_, *D*_*I*_ and *D*_*V*_ are their diffusion coefficients, *a* the tumor growth rate, *k* its carrying capacity, *k*_3_ the decay rate of free viruses, *t* the time and *r* the radial coordinate (assuming radial symmetry, as explained in detail below). Some authors [20] have argued that, in some situations, it may be assumed that $\frac {\partial \lbrack V]}{\partial t}=0$ and therefore, in homogeneous systems $\left (\frac {\partial ^{2}[V]}{\partial r^{2}}=0\right) $, Eq. (2) implies that $[\!V](r,t)=\frac {k_{2}Y}{k_{3}}[\!I](r,t)$. However, this assumption (free virus in steady-state) could only be applied if the decay rate of virus *k*_3_ is much larger than the decay rate of the infected cell population *k*_2_ [20]. From these arguments, they obtain the two-equation system used by Wodarz et al. [5], namely

(5) $$ \begin{aligned} \frac{\partial\lbrack \!T](r,t)}{\partial t} & =D_{T}\frac{\partial^{2}[\!T](r,t)}{\partial r^{2}}\\ &\quad +a[\!T](r,t)\left\{ 1-\frac{[\!I](r,t)+[\!T](r,t)}{k}\right\}\\ & \quad-b[\!I](r,t)[\!T](r,t), \end{aligned}  $$

(6) $$ \begin{aligned} \frac{\partial\lbrack \!I](r,t)}{\partial t} & =D_{I}\frac{\partial^{2}[\!I](r,t)}{\partial r^{2}}-k_{2}[\!I](r,t)\\ &\quad +b[\!I](r,t)[\!T](r,t), \end{aligned}  $$

where $b=\frac {k_{1}k_{2}Y}{k_{3}}$.

However, we find two drawbacks in the model (5)–(6) to explain our VSV-GBM system. First, Wodarz assumes $\frac {\partial \left [ V\right ] }{\partial t}=0$, and thus [*V*]∝[*I*]. As said before, this may be valid when *k*_3_≫*k*_2_ and in some non-spatial models [20] but this is in general not valid for the spatial propagation of virus infections. In such cases, at points located far away from the initially infected area, before the arrival of the infection front we have [*V*]=0, when the infection arrives [*V*]≠0, and after all viruses (and infected cells) have decayed, we have again [*V*]=0. Therefore, when dealing with spatial infection fronts we have $\frac {\partial \left [ V\right ] }{\partial t}=0$ only at early and late times, but $\frac {\partial \left [ V\right ] }{\partial t}>0$ when the first viruses arrive and $\frac {\partial \left [ V\right ] }{\partial t}<0$ after the passage of the infected front. Moreover, our experimental data (see “Parameter values” section) suggest that in our system *k*_3_ is very close to *k*_2_ and therefore, the assumption *k*_3_≫*k*_2_is not satisfied here either. Therefore, in contrast to Ref. [5], we cannot assume $\frac {\partial \left [ V\right ]}{\partial t}=0$, thus we deal with three differential equations (for viruses, susceptible tumoral cells, and infected tumoral cells).

Our second objection to the model (3)–(2) [and its simplification (5)–(6)] is that, according to the first reaction in Eq. (1), virus adsorption causes not only the same decrease in susceptible tumor cells [last term in Eq. (3)] as the increase in infected cells [last term in Eq. (4)], but also the same decrease in viruses. Thus a term −*k*_1_[ *V*](*r*,*t*)[ *T*](*r*,*t*) is missing in the right side of Eq. (2), in agreement with many previous works on virus infections [15–17, 21].

In the next section we develop a model which takes both points into account, as well as other important effects (namely, time-delay effects).

## Methods

### Mathematical models

**Fig. 1:**
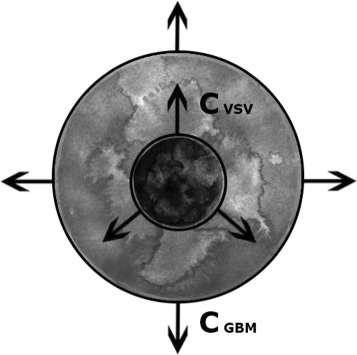
Two circles representing the two propagation fronts of VSV and GBM. A front of tumor cells spreads radially (*outer circle*). After some time, viruses are inoculated at the center, and a virus front spreads (*inner circle*). If the inner circle expands faster than the outer one (*c*_*VSV*_>*c*_*GBM*_), the viruses will eliminate the tumor

Here we want to develop a simple, but complete model to understand the dynamics of a virus-tumor system. The theoretical model should be able to explain an in vitro experiment where a virus injected into the center of a tumor spreads through the tumor cell population in a basically two-dimensional geometry. Therefore, we can think of the virus-tumor system as formed by two fronts of propagation, which could be represented as two concentric circles if we assume radial symmetry. The diagram in Fig. 1 illustrates this idea. The outer circle represents the tumor cells, which spread to the outside through a non-specific medium. The inner circle represents the viruses spreading within the tumor. Viruses diffuse through the medium before infecting tumor cells. When infected cells die, a new generation of viruses is created and the process begins anew.

The main idea and experimental laboratory data come from Ref. [9], where Wollmann et al. compare nine types of viruses with strong oncolytic potential and conclude that four of them, VSV included, would be worthy of more rigorous studies. Because in subsequent papers [11, 22] they worked with VSV and its recombinant variants or strains, we decided to focus solely on VSV and use these data as experimental basis.

Below we present three increasingly complete (and complicated) models.

#### Model 1

As a first approach, we adapt the model by Wodarz et al. [5] to the conditions in our VSV-GBM systems, i.e., we do not assume $\frac {dV}{dt}=0$, and therefore [ *V*] is not proportional to [ *I*] and we need to include the virus dynamics explicitly in the model.

Now the evolution of the virus-tumor system is described by

(7) $$ \frac{\partial\lbrack \!V](r,t)}{\partial t}=D_{VSV}\frac{\partial^{2} [\!V](r,t)}{\partial r^{2}}+F(r,t),  $$

(8) $$ \begin{aligned} \frac{\partial\lbrack \!T](r,t)}{\partial t} & =D_{GBM}\frac{\partial^{2}[\!T](r,t)}{\partial r^{2}}\\ &\quad +a[\!T](r,t)\left\{ 1-\frac{[I](r,t)+[\!T](r,t)}{k}\right\}\\ &\quad -k_{1}[\!V](r,t)[\!T](r,t), \end{aligned}  $$

(9) $$ \frac{\partial\lbrack \!I](r,t)}{\partial t}=k_{1}[\!V](r,t)[\!T](r,t)-k_{2} [I](r,t).  $$

The first equation describes the evolution of the virus population over time. The viruses can spread ruled by the diffusion coefficient *D*_*VSV*_ and the Laplacian (or second space derivative). The function *F*(*r*,*t*) in Eq. (7) incorporates all processes of infection, replication and death and is defined by

(10) $$ \begin{aligned} F(r,t) & =-k_{1}[\!V](r,t)[\!T](r,t)\\ &\quad +k_{2}Y[\!I](r,t)-k_{3}[\!V](r,t). \end{aligned}  $$

Note that the first term was not included in the models by Nowak-May and Wodarz [Eq. (2)] (see our second objection in “Previous mathematical approaches” section).

Equation (8) describes the change in the number of tumor cells over time. Similarly to viruses, glioblastoma cells can also move, characterized by their own diffusion coefficient *D*_*GBM*_.

Finally, Eq. (9) represents the evolution of infected tumoral cells. We assume that these cells do not move, in agreement Fig. 3D of Ref. [9], where the experiment shows how the infected cells (U-87 MG glioblastoma cells) initially introduced do not move through the host layer throughout the observation period.

#### Model 2

As we shall see in “Results and discussion” section, model 1 needs further improvements. In model 2 we take into account that infected tumoral cells do not die instantaneously, instead there is a time delay before the cell dies and releases the new progeny of viruses. We will denote this delay or eclipse time as *τ* and include it into the terms related to the death of infected cells. Thus infected cells will not die proportionally to the density of infected cells at the present time, *k*_2_[*I*](*r*,*t*), but proportionally to the density of infected cells at a previous instant *t*−*τ*, *k*_2_[*I*](*r*,*t*−*τ*), to properly include this time delay effect on the decay process. It has been shown that the term −*k*_2_[*I*](*r*,*t*−*τ*) agrees well with experimental data in a different context (infections of non-tumor cells) [23]. Other reaction-diffusion models do also apply *t*−*τ*, although in an alternative way [24, 25]. The differences between their approach and ours is analyzed in Ref. [23].

Therefore, when introducing the delay in the death of infected cells, Eqs. (9) and (10) are modified directly and Eq. (7) changes because the function *F*(*r*,*t*), Eq. (14), is also modified. We do not modify the growth term in Eq. (8) because the reproduction of tumoral cells depends on the total number of tumor cells (infected and susceptible) at that precise instant *t*. So, we consider the model

(11) $$ \frac{\partial\lbrack \!V](r,t)}{\partial t}=D_{VSV}\frac{\partial^{2} [\!V](r,t)}{\partial r^{2}}+F(r,t),   $$

(12) $$ \begin{aligned} \frac{\partial\lbrack \!T](r,t)}{\partial t} & =D_{GBM}\frac{\partial^{2}[\!T](r,t)}{\partial r^{2}}\\ &\quad +a[\!T](r,t)\left\{ 1-\frac{[\!I](r,t)+[\!T](r,t)}{k}\right\}\\ &\quad -k_{1}[\!V](r,t)[\!T](r,t), \end{aligned}  $$

(13) $$ \frac{\partial\lbrack \!I](r,t)}{\partial t}=k_{1}[\!V](r,t)[\!T](r,t)-k_{2} [\!I](r,t-\tau),   $$

where now

(14) $$ \begin{aligned} F(r,t) & =-k_{1}[\!V](r,t)[\!T](r,t)\\ &\quad +k_{2}Y[\!I](r,t-\tau)-k_{3}[\!V](r,t). \end{aligned}  $$

This second model is, actually, an approximation of our next model (see Model 3 below).

#### Model 3

Model 2 takes into account a delay time in the reactive process *I*→*Y*·*V*, but here we shall see that the delay time also has a very important diffusive effect. The diffusion dynamics of the virus concentration in Eq. (11) is Fickian, which means that it does not take into account the effect of the time delay *τ*. In year 2002 it was shown [26] that it is very important to take into account that *τ* is the time interval during which a virus does not move in space (because it is inside an infected cell), thus the delay time should affect the model by slowing down the spread of viruses. Therefore it is necessary to incorporate also this effect to reach a realistic model. For this reason, Eq. (11) must be replaced by an equation with second-order terms to include this diffusive time-delay effect [17, 26, 27].

Thus, finally we describe the spatial-time dynamics of the whole system with the following equations:

(15) $$ \begin{aligned} \frac{\partial\lbrack \!V](r,t)}{\partial t}&+\frac{\tau}{2}\frac{\partial^{2}[\!V](r,t)}{\partial t^{2}}=D_{VSV}\frac{\partial^{2}[\!V](r,t)}{\partial r^{2}}\\ &+F(r,t)+\frac{\tau}{2}\left. \frac{\partial F(r,t)}{\partial t}\right\vert_{g}, \end{aligned}  $$

(16) $$ \begin{aligned} \frac{\partial\lbrack \!T](r,t)}{\partial t} & =D_{GBM}\frac{\partial^{2}[\!T](r,t)}{\partial r^{2}}\\ &\quad +a[\!T](r,t)\left\{ 1-\frac{[\!I](r,t)+[\!T](r,t)}{k}\right\}\\ &\quad -k_{1}[\!V](r,t)[\!T](r,t), \end{aligned}  $$

(17) $$ \frac{\partial\lbrack \!I](r,t)}{\partial t}=k_{1}[\!V](r,t)[\!T](r,t)-k_{2} [\!I](r,t-\tau),  $$

where the terms proportional to *τ* in Eq. (15) are the new, second-order terms. A self-contained derivation of Eq. (15) can be found in Ref. [23], Appendix A.

In Eq. (15) *F*(*r*,*t*) is again given by Eq. (14), and Eqs. (12) and (13) from model 2 remain unchanged (Eqs. (16) and (17), respectively).

Note that *F*(*r*,*t*) can be understood as the variation of [*V*] over time due to all reactive processes, but not to diffusive processes, i.e. $F(r,t)=\left. \frac {\partial \lbrack V](r,t)}{\partial t}\right \vert _{g}$. This allows the proper calculation of the first time derivative as [17, 27]

(18) $$ \begin{aligned} \left. \frac{\partial F(r,t)}{\partial t}\right\vert_{g} & =-k_{1} F(r,t)[\!T](r,t)-k_{1}[\!V](r,t)\frac{\partial\lbrack \!T](r,t)}{\partial t}\\ &\quad +k_{2}Y\frac{\partial\lbrack \!I](r,t-\tau)}{\partial t}-k_{3}F(r,t). \end{aligned}  $$

For systems in which the infected cells diffuse appreciably (not our case, see the last paragraph in the model 1 section), an age-structure model with this additional diffusive-delay effect has been proposed by Gourley and Kuang in Ref. [24], p. 558.

In the equation describing the virus dynamics, Eq. (15), we include corrections only up to second order [17, 27]. It has been shown in previous work [26] that the divergence between second-order approximation and full time-delayed equations is small, and thus we can exclude terms of higher orders.

### Front speeds

#### Virus front

Using models 1–3 above, we look for realistic travelling-wave speeds for both the propagation front of viruses (inner front, Fig. 1) and the propagation front of tumor cells (outer front, Fig. 1). Finding the propagation speeds will allow us to compare to the in vitro experiments in order to validate our approach.

In all models 1–3, we can transform the problem into a single-variable system by using the co-moving coordinate *z*=*r*−*ct*. Like in previous works [15, 26], we assume the concentration of the three populations at the leading edge of the moving front (*z*→*∞*) can be written as [ *T*]=*k*−*ε*_*T*_· exp(−*λz*), [*I*]=*ε*_*I*_· exp(−*λz*) and [ *V*]=*ε*_*V*_· exp(−*λz*), thus we assume that tumoral cells are nearly at maximum concentration at large distances from the inoculation point of the viruses, while viruses and infected cells are barely present. We make use of this transformation because beyond the edge of the front of infected cells and viruses, there is only a continuous medium of tumor cells. For non-trivial solutions to exist, the determinant of the matrix corresponding to the linearized model must be zero. The characteristic equations for models 1, 2 and 3 are, respectively,

(19) $$ \begin{aligned} \left(\lambda c+k_{2}\right) & \left(\lambda c-D_{VSV}\lambda^{2}+kk_{1}+k_{3}\right)\\ - & kk_{1}k_{2}Y=0, \end{aligned}  $$

(20) $$ \begin{aligned} \left(\lambda c+k_{2}\mathrm{e}^{-\lambda c\tau}\right) & \left(\lambda c-D_{VSV}\lambda^{2}+kk_{1}+k_{3}\right)\\ - & kk_{1}k_{2}Y\mathrm{e}^{-\lambda c\tau}=0, \end{aligned}  $$

(21) $$ \begin{aligned} \left(\lambda c+k_{2}\mathrm{e}^{-\lambda c\tau}\right) & \left[ \lambda c-D_{VSV}\lambda^{2}+kk_{1}+k_{3}\right.\\ & \left. +\frac{\tau}{2}\left(\lambda^{2}c^{2}-k^{2}{k_{1}^{2}}-2kk_{1} k_{3}-{k_{3}^{2}}\right) \right] \\ -kk_{1}k_{2}Y\mathrm{e}^{-\lambda c\tau} & \left[ 1+\frac{\tau}{2}\left(\lambda c-kk_{1}-k_{3}\right) \right] =0. \end{aligned}  $$

According to marginal stability analysis [28], the propagation front moves with the minimum possible speed. Therefore,

(22) $$ c_{VSV}=\underset{\lambda>0}{\min}\left[ c\left(\lambda\right) \right],   $$

where *c*(*λ*) is given implicitly by Eqs. (19), (20) and (21). From Eq. (22) we can numerically estimate the speed of VSV infection.

The resulting propagation speeds for models 1–3 will be calculated and plotted in “Results and discussion” section.

We also solve the third model by numerical integration and find the front speed from the position of the virus front wave in a successive steps of time.

#### Glioblastoma front

Under the hypothesis of two propagation fronts, as shown in Fig. 1, the outermost front would correspond the tumor cells, [*T*] (GBM in our case of study). In the conditions near this front, all models can be greatly simplified since here the populations of viruses and infected cells are zero (see the outer circle in Fig. 1 for a better understanding), so [ *V*](*r*,*t*)=0 and [ *I*](*r*,*t*)=0. Hence, it is only necessary to work with the equation for the tumoral cells, Eq. (16) for example, but remembering that [ *V*](*r*,*t*)=[ *I*](*r*,*t*)=0,

(23) $$ \begin{aligned} \frac{\partial\lbrack \!T](r,t)}{\partial t} & =D_{GBM}\frac{\partial^{2}[\!T](r,t)}{\partial r^{2}}\\ &\quad +a[\!T](r,t)\left\{ 1-\frac{[\!T](r,t)}{k}\right\}. \end{aligned}  $$

At the leading edge of this front, we assume that [ *T*](*r*,*t*)=*ε*_*T*_· exp(−*λz*), and after some algebra we easily obtain the speed of the glioblastoma front,

(24) $$ c_{GBM}=2\sqrt{D_{GBM}\text{\thinspace}a},   $$

where *D*_*GBM*_ is the glioblastoma diffusion coefficient and *a* the growth rate, both estimated in the next subsection. Note that Eq. (24) is the well-known Fisher propagation speed [29]. Some recent extensions have been proposed [6, 30], but they are not necessary for the purposes of the present paper.

### Parameter values

We estimate most of our parameters from in vitro experiments on VSV applied to GBM [9, 11, 22]. The parameters that we could not draw from such experiments have been obtained from other rigorous studies on VSV or glioblastoma.

We use two different values of *D*_*VSV*_ because the diffusion coefficient of VSV has not been measured in gliomas. The only value of VSV available (measured in an specific water solution) is *D*_*VSV*_=8.37·10^−5^ cm^2^/h [31]. Another value measured in agar of VSV-similar viruses is *D*_*VSV*_=1.44·10^−4^ cm^2^/h [17].

Concerning *D*_*GBM*_, Stein et al. [32] performed an in vitro experiment in which a GBM tumor spheroid is implanted into a collagen gel. The diffusion coefficient is measured by tracking individual cells on the first day, calculating their motion and averaging over many cells. Stein and co-workers measure diffusion coefficients in the radial and angular directions, which lead to the value *D*_*GBM*_=3.75·10^−6^ cm^2^/h [6].

Besides spreading, the number of cells also increases. The parameter *a* is the corresponding proliferation rate. In vitro measurements provide ample scope for this parameter, 0.04<*a*<0.3 day ^−1^ [33], and similarly in vivo studies yield 0.01<*a*<0.14 day ^−1^ [34].

The saturation cell density, *k*, measures the maximum concentration of tumor cells (susceptible and infected) per unit volume that the system can support, and its usual value is *k*=10^6^ cells/cm^3^ (e.g.,Refs. [35, 36]).

We next analyze the rest of parameters, which are calculated from the experimental studies by Wollmann et al. [9, 11, 22].

The yield or burst size *Y* represents the total amount of viruses produced by the death of a single infected cell. There is no accurate numerical value calculated for the case of VSV infecting GBM. However, by studying Fig. 4 in Ref. [11] we can obtain an estimation. The burst size can be understood as the ratio between the maximum and initial number of viruses, i.e. $Y=\frac {V_{\max }}{V_{0}}$. From that figure, *V*_0_ is between 10−100 PFU/ml (last two plots in Fig. 4 in [11]) and *V*_max_ between 10^8^−10^9^ PFU/ml (the maximum is reached between 1 and 2 days post infection), so we conclude that 10^6^<*Y*<10^8^. This also agrees with the value measured in Ref. [37], although in that case VSV infects BHK-21 cells (not GBM cells).

We have seen that there is a time lapse between a cell being infected by a virus and that cell dying (and therefore, adding more viruses to the system). This time lapse is called the delay time, *τ*. It plays a main role in the virus propagation speed, but has not been accurately measured. From the in vitro experiments described in Ref. [9] we can try to estimate the value of *τ*. On one hand, we know that the death of infected cells begins about 6 hours post infection (hpi) of the virus to susceptible tumoral cells. We also know that infected cells can be seen as early as 4 hpi (they are tracked down using GFP fluorescence). From both data, we conclude that viruses leave infected cells at least 2 h after infection. On the other hand, in a different experiment infected cells are added directly (rather than infecting viruses) and new infected cells were detected after 12 h. This period includes the time needed for the viruses to multiply within the infected cells, leave the cell and infect new tumoral cells. So we can also assume that *τ* must be lower than 12 h. In summary, we will work with the range 2<*τ*<12 h.

The adsorption rate, *k*_1_, describes the efficacy of the whole infection process (rate of virus entry and probability of successful infection). The value of *k*_1_ could be measured in an experiment where the reproduction of viruses and host cells were prevented. Such an experiment has been performed for other viruses [38] but not for VSV infecting GBM. Since we do not have the ideal conditions in the experiments cited before [9, 11, 22], we will use the earliest data post-inoculation available in the experimental data in Ref. [11] to minimize the effect of reproduction and thus obtain the best possible estimation for *k*_1_.

Equations (7) and (8) are simplified in the absence of reproduction and natural death, and when the population is studied as a whole (i.e. ignoring diffusion terms) we have

(25) $$ \frac{d[\!V](t)}{dt}=\frac{d[\!T](t)}{dt}=-k_{1}[\!V](t)[\!T](t).   $$

Obviously, integrating we get [ *T*](*t*)= [ *V*](*t*)+*ξ*, where *ξ* is the constant of integration. Note that *ξ* is the difference between the concentrations of tumor cells and viruses. In order to estimate *k*_1_, we can rewrite the previous Eq. (25) as $\frac {d[T](t)}{dt}=-k_{1}[\!T](t)\left ([\!T](t)-\xi \right) $ and making the necessary algebra we obtain the final formula for calculating the adsorption rate,

(26) $$ k_{1}=\frac{1}{\xi\left(t-t_{0}\right) }\left[ \ln\left(\frac{T}{T-\xi }\right) -\ln\left(\frac{T_{0}}{T_{0}-\xi}\right) \right].  $$

It is difficult to know the exact concentration of cells at the beginning of the experiment or at certain time *t*, because only relative concentrations were reported. However, extrapolating data provided in the previous cited papers by Wollmann et al. (Fig. 3C Control in [11], bar G/GFP), we believe it is correct to assume that the values of initial tumor cells lie in the range *T*_0_=10^6^−10^8^ cells/cm^3^, and that *T*=0.65*T*_0_ cells/cm^3^, *t*−*t*_0_=36 h. This allows the calculation of the adsorption rate, as 5·10^−10^<*k*_1_<5·10^−8^ cm^3^/h. This is a rather wide range, but we show in “Effect of *k*_1_ and *Y*” section that *k*_1_ (as well as *Y*) does not overly affect the propagation front speed of VSV.

Finally, parameters *k*_2_ and *k*_3_ correspond to the rates of death of infected cells and virus, respectively. Therefore, the average life-time of an infected cell and a virus are 1/*k*_2_ and 1/*k*_3_, respectively.

The rate of death of infected cells *k*_2_ could be also understood as the growth of viruses. Thus, for *t*<*τ* no new virus are seen in the corresponding experiment (because no infected cell has died yet), but for *t*≥*τ* the infected cells start to die ruled by *dI*=−*k*_2_*I*_0_*dt*. The death of each infected cell produces *Y* virus, thus *dV*=−*YdI*=*k*_2_*YI*_0_*dt*=*k*_2_*V*_max_*dt*. Integrating, we get $k_{2}=\frac {V_{\max }-V_{0} }{\Delta t\cdot V_{\max }}\approx \frac {1}{\Delta t}=\frac {1}{t^{\ast }-\tau }$, where *t*^∗^ represents the time when the virus population reaches its maximum. According to Fig. 4B in Ref. [11], experimental data (labeled as VSV-G/GFP) show that the maximum is reached at *t*^∗^=(48±12) h. Nevertheless, the final result of *k*_2_ will depend on *τ* and we have a range rather than a single value for *τ* (see above). Note, however, that for model 1 there is no time delay, so *k*_2_ is calculated straightforwardly as the inverse of time *t*^∗^ at which the concentration of viruses reaches its maximum, $k_{2}=\frac {1}{t^{\ast }}$ h ^−1^. Models 2 and 3 are dealt with in “Results and discussion” section.

The evolution of the viruses over time in an environment where they die but cannot reproduce is ruled by *dV*=−*k*_3_*Vdt*. Through simple integration we get *V*(*t*)=*V*_0_ exp[−*k*_3_(*t*−*t*_0_)]. In the same experiment as before, Fig. 4B in Ref. [11], we now have two cases where these conditions are exactly reproduced (because VSV-dG-GFP and VSV-dG-RFP are replication-restricted virus variants, so they basically die). We can estimate both values of *k*_3_ from the experimental data, namely *V*(*t*=24 h)=30 PFU/cm^3^, *V*(*t*=48 h)=20 PFU/cm^3^ and *V*(*t*=72 h)=8 PFU/cm^3^ for the mutant dG-GFP and *V*(*t*=24 h)=12 PFU/cm^3^, *V*(*t*=48 h)=8 PFU/cm^3^ and *V*(*t*=72 h)=6 PFU/cm^3^ for dG-RFP. Performing linear fits to ln*V* versus *t*, we obtain that 0.014<*k*_3_<0.028 h ^−1^.

## Results and discussion

### GBM and VSV front speeds: theory versus experiment

Our main objective is to obtain realistic values for the propagation speeds in an in vitro virus-tumor system, providing positive results from a biophysical point of view for the realization of these treatments.

In “Methods” section we have described three possible models for our VSV-GBM system and the necessary experimental parameter values. Here we present the speeds predicted by these models.

The case of tumor expansion has a single, simple solution for all models, Eq. (24), since the infection does not play a role here. Substituting the values of *D*_*GBM*_ and *a* we obtain that *c*_*GBM*_=2.5·10^−4^ cm/h, with *a*=0.1 day ^−1^, which we think is a reasonable mean value. Indeed, the range of measurements of the proliferation rate is 0.01<*a*<0.3 day ^−1^, which yields a range of speeds 7.9·10^−5^<*c*_*GBM*_<4.33·10^−4^ cm/h). Stein and co-workers measured an experimental in vitro speed range of 2.37·10^−4^<*c*_*GBM*_<5.54·10^−4^ cm/h [33], which is consistent with our model, despite the simplicity of Eq. (24).

The case of the virus front is less straightforward. As we have already discussed in “Parameter values” section, a very important but not strictly well-measured parameter is the delay time *τ*. Therefore, the speed results have been calculated in terms of this parameter, *c*(*τ*). The death rate of infected cells *k*_2_ also changes, because it depends directly on *τ*.

**Fig. 2:**
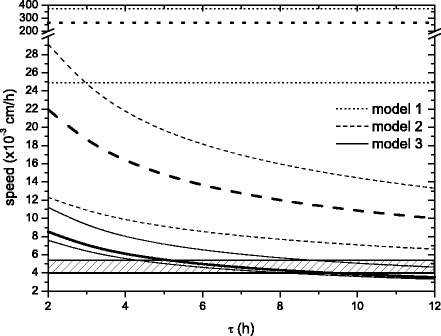
VSV front propagation speed as a function of the delay time *τ*, for model 1 (dotted lines), model 2 (dashed curves) and model 3 (solid curves). The hatched area shows the experimental in vitro VSV front speed [9]. Upper bounds are computed from: *k*_1_=5·10^−8^ cm^3^/h, $k_{2}=\frac {1}{36-\tau }$ h ^−1^ ($k_{2}=\frac {1}{36}$ h ^−1^ for model 1), *k*_3_=0.014 h ^−1^, *Y*=10^8^ and *D*_*VSV*_=1.44·10^−4^ cm^2^/h. Lower bounds are computed from: *k*_1_=5·10^−10^ cm^3^/h, $k_{2}=\frac {1}{60-\tau }$ h ^−1^ ($k_{2} =\frac {1}{60}$ h ^−1^ for model 1), *k*_3_=0.028 h ^−1^, *Y*=10^6^ and *D*_*VSV*_=8.37·10^−5^ cm^2^/h. The results from typical values (bold lines) are computed from: *k*_1_=5·10^−8^ cm^3^/h, $k_{2}=\frac {1}{48-\tau }$ h ^−1^ ($k_{2}=\frac {1}{48}$ h ^−1^ for model 1), *k*_3_=0.02 h ^−1^, *Y*=10^8^ and *D*_*VSV*_=8.37·10^−5^ cm^2^/h. In all the cases *k*=10^6^ cells/cm^3^

The infection front speed, *c*_*VSV*_, can be seen in Fig. 2. For each of the 3 models we have plotted the results from typical parameter values (bold lines). To compute these results we have chosen the parameter values that seem to be the most representative and accepted for this experiment: average values of *k*_2_ and *k*_3_, the value of *D*_*VSV*_ calculated for VSV in an specific water solution and the larger values of *k*_1_ and *Y*. However we have also computed *c*_*VSV*_ by varying each of the parameters of Eqs. (19)–(21), with the exception of *k* because *k*=10^6^ cells/cm^3^ is a widely accepted value in research papers (see “Parameter values” section). In Fig. 2 we include the upper and lower bounds for the front speed obtained, for each of the 3 models, from the experimental parameter ranges (parameter values are specified at the caption).

**Fig. 3:**
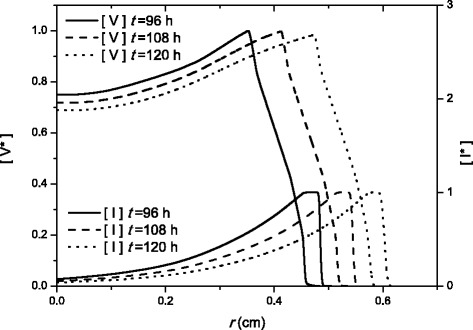
Radial profiles of [*V* ^∗^] and [*I* ^∗^] at three different times for model 3. The labels of *V* ^∗^ and *I* ^∗^ stand for the units used, defined as $\frac {\left [ V\right ]}{\left [ V\right ]_{\max }}$ and $\frac {\left [ I\right ] }{\left [ I\right ]_{\max }}$, respectively. The profiles are computed from numerical integration

The hatched area in Fig. 2 corresponds to the experimental values of VSV speed estimated from the in vitro experiment by Wollmann et al. in Ref. [9], Fig. 3A.

Dotted lines correspond to the analytical results to model 1, Eqs. (7)–(10), i.e. the classical model adapted from the equations in Ref. [5]. Obviously they are horizontal lines, since they do not depend on *τ*. As we can see in Fig. 2, model 1 yields speeds much faster than the experimental observations. The curves are the numerical results from our time-delayed reaction-diffusion models. Dashed curves correspond to model 2, given by Eqs. (11)–(14). We see that just by taking into account the eclipse or delay time on the death of infected cells, we obtain much better results as compared with experimental velocities, although not enough to satisfactorily explain the data (the minimum bound of model 2 in Fig. 2 is above the hatched area). Finally, solid curves in Fig. 2 correspond to model 3 (please recall that this is extremely close to the full time-delayed equation, see “Methods” section). The equations for this main model, Eqs. (15)–(18), when considering typical parameter values, produce results that agree with the experimental data within a range of *τ* between 5.0 and 9.3 h.

According to our best description (model 3), the entire range of speed *c*_*VSV*_ in Fig. 2 is an order of magnitude faster than the speed of propagation of glioblastoma, *c*_*GBM*_, (see above). Therefore the virus front could theoretically reach the tumoral front and infect it all. We stress that this is a model appropriate for in vitro experiments, whereas in vivo more complex models will be necessary (as discussed below).

In Fig. 3 we show snapshots of the viruses and infected cells profiles as functions of the radial axis, computed from the computational simulations at three time instants. The simulations have been performed by numerical integration of model 3, which is biologically more realistic and produces results in agreement with the experimental data (see Fig. 2). We use the typical parameter values used in Fig. 2 (bold lines, see caption for the values). We can see in Fig. 3 that both propagation fronts advance at the same speed and with regular shapes.

From the profiles we can see that the number of infected cells grows rapidly, then there is a plateau of infected cells (as a result of the time delay *τ* before any infected cell dies), and then decay at a rate *k*_2_. The virus profiles show an abrupt rise when infected cells start dying (end of the plateau of infected cells) and then keep rising up to a peak. Behind this peak, the virus death term *k*_3_ predominates over the virus production, and the number of viruses decay. Although Fig. 3 seems to indicate that the front of infected cells appears prior to the virus front, the opposite happens (this can be appreciated by enlarging the vertical scale).

From these simulations we can calculate the front speed by tracking the position of the edge of the front of the virus at successive steps of time. A simple space vs time data is generated and then, the front speed is directly the slope. From the simulations (parameter values are the same than typical values in Fig. 2 with *τ*=6 h) we find a front speed of 4.829·10^−3^ cm/h. The relative error between the simulations and the analytic speed [ *c*_*VSV*_=4.853·10^−3^ cm/s, from Eqs. (21)–(22)] is only about 0.5 *%*.

An alternative way to know the front propagation speed from Fig. 3 is the plateau of infected cells. Its width is directly related with the time delay *τ* and the infection front speed as *width*=*τ*·*c*. Then, the result for the speed is (0.53858−0.51317) cm / 6 h =4.735·10^−3^ cm/h (distances for *t*=108 h), and the relative error (compared with the analytical results with same parameter values than the simulations) is lower than 2.5 *%* (*c*_*VSV*_=4.853·10^−3^ cm/s).

### Effect of *k*_1_ and *Y*

In “Parameter values” section we have estimated the values of the parameters used in our mathematical models. Some of them, e.g. *D*_*VSV*_, *D*_*GBM*_ and *k*, have well-defined values, which are taken from the references indicated in the text. The delay time *τ* plays a very important role and therefore we have found the front propagation speed as a function of this parameter (remember that $k_{2}=\frac {1}{48-\tau }$, so we could add *k*_2_ to this argument). Other parameters like *a* and *k*_3_ have a range of possible values, albeit a narrow one, and as such we use the mean value, or that usually accepted by other sources. Lastly, parameters *Y* and *k*_1_ have very wide ranges, spanning several orders of magnitude, but as we shall show below, they do not have an important effect on the virus front speed.

**Fig. 4:**
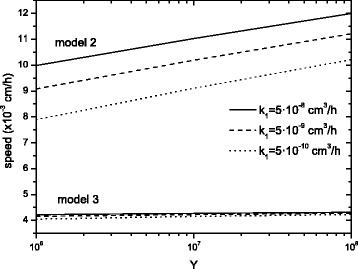
VSV invasion speed on GBM for various values of *Y* and *k*_1_. The other parameter values are *k*=10^6^ cm ^−3^, $k_{2}=\frac {1}{40}$ h ^−1^, *τ*=8 h and *k*_3_=0.02 h ^−1^. Model 3 proves that neither *Y* nor *k*_1_ affect much the speed of the front

In Fig. 4 the speed of VSV is calculated from model 2 (Eqs. (11)–(14)) and model 3 (Eqs. (15)–(18)). Setting the typical parameter values previously used in Fig. 2 (bold curves) and Fig. 3 for *D*_*VSV*_, *D*_*GBM*_, *k*, *k*_3_ and the average value *τ*=8 h (so *k*_2_=1/40 h ^−1^), which yields results consistent with the range of experimental speeds (Fig. 2), we have varied the values of *Y* and *k*_1_ for each of both models.

In model 2 (upper curves in Fig. 4) the speed dependence on *Y* and *k*_1_ is fairly important. Indeed, by increasing these variables by two orders of magnitude, the speed increases on average by 25 and 18 %, respectively. However, looking at the best approach, model 3 (lower curves), we note that the speed increases only by 3 and 2 % for *Y* and *k*_1_, respectively.

Therefore, model 3 has little dependence on the parameters *Y* and *k*_1_ and the delay time is the most important parameter (Fig. 2). In contrast, model 2 depends more directly on both parameters, although *τ* still remains the crucial one (compare the change of the speed in Fig. 2 with those in Fig. 4 for model 2). To obtain a speed of virus propagation similar to the observed data (*c*≈5·10^−3^ cm/h) with model 2, we should modify *Y* and *k*_1_ out of the experimental ranges. Indeed, their values should be about *Y*=10^4^ or *k*_1_=5·10^−12^ cm^3^/h. Therefore, we could get a speed in agreement with the experimental data, but only using unrealistic parameter values, which do not correspond to VSV. This is further proof that our final model 3, the time-delayed reaction-diffusion set of equations, is a good mathematical tool to explain this kind of virus-tumor biological systems.

## Conclusions

A simple set of time-delayed equations have been built to understand the dynamics of a virus-tumor system. We have improved a previous model with new ideas and carefully incorporated experimental results (especially Ref. [9]). Figure 2 proofs that our best framework (model 3) is in reasonable agreement with the experimental data. Furthermore, the figure shows that neither model 1 nor model 2 can explain the experimental data. So it is absolutely necessary to add the second-order terms proportional to *τ* in Eq. (15) to properly include the time-delay effect.

We have shown that the delay time *τ* is the crucial parameter in our models (even when compared to other parameters that are strongly unknown, such as *k*_1_ and *Y*). As we could have expected, as *τ* increases, the speed of the virus front decreases, because viruses spend more time inside the cell, and therefore at rest. In spite of being of utmost importance, the role of the delay or eclipse time has not been taken into account in previous models of virus treatment of tumors [5, 18, 19].

We have found that our new model can satisfactorily predict the front speed for the lytic action of oncolytic VSV on glioblastoma observed in vitro. But this is only a first step towards a deep biophysical understanding of the principles of virus-tumor space-time spread in a complex system. This model could be extended to be applied to in vivo experiments where, among other effects, the immune response should be also included in the model because it may play a significant role regulating the efficacy of the therapy. In particular, it seems that there is currently no agreement about which approach is better in oncolytic therapy, whether to modify oncolytic viruses to obtain the maximum antitumoral immune response [39], to transiently suppress the immune response [40], or to use a combination of both [40]; future appropriate modeling of the three scenarios might help in tackling this controversy from a different perspective.

In this paper we have focused on GBMs because of the experimental data available, but our model could apply also to many non-diffusive cancers, for which viral therapy is a promising approach [18, 19, 41], since the reaction-diffusion equations for the viruses [Eqs. (15)–(18)] will still be valid, even though in such cases tumor cells will not diffuse. Thus, we provide a basis that can be applied in the near future to realistically simulate in vivo virus treatments of several cancers.

## Reviewers’ comments

### Reviewer’s report 1

Yang Kuang, Arizona State University, United States of America

**Reviewer comments:** The paper is mostly well written with only a few places where I can suggest the authors to consider adding more details or be aware of alternative explanations.

1: The authors made a valid point that $\frac {\partial \left [ V\right ] }{\partial t}$ is not always close to 0. However, a routine argument used in the mathematical modeling community is the quasi-state-steady approximation. This argument suggests that due to virus’ fast dynamics (virus reproduces probably in less than one hour once the first virus reproduced), over the longer period tumor cell growth time (of days), on average, the total virus amount changes at a rate far less than the maximum possible rate when all viruses reproduce at the maximum rate. Mathematically, one can show this quasi-steady-state level can be approximated by setting $\frac {\partial \left [ V\right ] }{\partial t}=0$ and solving *V* in terms of other variables. 2: A better reference in the virus modeling context for the need of adding the virus loss term −*k*_1_[ *V*](*r*,*t*)[*T*](*r*,*t*) may be E. Beretta and Y. Kuang: Modeling and analysis of a marine bacteriophage infection. Math. Biosc. 149, 5776(1998), where each and every term is carefully explained in the context of biology. 3: The justification for the Eq. (15) is mathematically simple, but mechanistically very ad hoc and difficult to follow. A possible alternative way to modeling the delay dependence of the diffusive action is to assign virus an age. A good reference on this approach is S. A. Gourley and Y. Kuang: A Delay Reaction-Diffusion Model of the Spread of Bacteriophage Infection, SIAM J. Appl. Math., 65, 50566(2005). 4: I think readers will benefit if the authors can provide more about the data nature and even a figure which may suggest that the VSV front is as described in Fig. 2. The authors may take a look of our recent work on in vitro GBM modeling and wave speed estimation to see how we handled this. Tracy L. Stepien, Erica M. Rutter, and Yang Kuang, 2015. A data-motivated density-dependent diffusion model of in vitro glioblastoma growth, Math. Biosc. Eng., 12, 11571172.

**Authors’ response:***We want to thank Dr. Y. Kuang for his revision of our manuscript and the suggestions provided to make it more complete and comprehensive. We answer each of his four comments separately below:*

*1. The quasi-state-steady approximation is truly widely used in mathematical modeling. It implies that the virus dies in a very short time, and the rate of the virus producing infected cells is short enough not to create a great amount of viruses. Mathematically, this means that,k*_3_≫*k*_2_ [20].*This condition is not fulfilled in our VSV-GBM system, wherek*_3_≈*k*_2_. *As a result, we consider that, in such a system, it is better to develop our model and perform the calculation with all three equations. We explain this before Eqs. (*5*) and (*6).

*2. We have added the relevant reference suggested at the end of “*Previous mathematical approaches*” section.*

*3. The justification of Eq. (*15*) is described in more detail in Ref. [*23*], Appendix A. We mention this below Eq. (*17*). We also cite [below Eq. (*18*)] the interesting reference suggested, which applies to systems in which infected cells diffuse (not our case).*

*4. A new figure (Fig. *3*) has been added to the paper showing the evolution in space and time of the concentration of virus and infected cell populations. We have computed these profiles through numerical integration and they now provide a new source from where to calculate the front speed for model 3. We see that this new value agrees with the experimental data found in Wollmann et al. experiments and with our analytical results. Readers will probably benefit from this new approach in order to completely understand the significance of our new equations and the good agreement between theoretical and experimental data. So, we specially appreciate the advice (from both referees) to include this kind of results.*

### Reviewer’s report 2

Georg Luebeck, Fred Hutchinson Cancer Research Center, United States of America

**Reviewer comments:** The mathematical framework presented by de Rioja et al. for oncolytic infection of GBM cells by the VSV virus and its impact on tumor growth in culture builds upon previous modeling. The authors show, convincingly (at least for the infection experiments in GBM cells), that it is important to include a time delay that represents the time from infection to cell death and production of new viral particles. Furthermore, the case is made that the time delay effect and sequestration of the virus in the infected cells leads to second order effects which further slow the spread of the virus.

Although the model is rather simplistic (it has radial symmetry, no vasculature, infection starting from a single point) and most kinetic rates are only known imprecisely, the agreement of the model prediction with the experimental data on the front speed of the VSV action is reassuring that the mathematical description of the augmented model is biologically plausible. The conclusions, of course, would have been stronger had the authors used an independent experimental model to validate their finding. Also, there is no notion of uncertainty in the predictions shown in Fig. 2. It would be useful if a sensitivity analysis could be included to demonstrate that model 3 is indeed the only model (among the 3) that is consistent with the experimental data.

Also, it is somewhat surprising that the authors did not also visualize the solutions of their models as radial density ‘snapshots’ at various endpoints. This (together with the parameter values used) could help others to reproduce their results.

**Authors’ response:***We thank the review by Dr. G. Luebeck, which is quite positive with our research manuscript. We have reviewed the text according to his suggestions.*

*As suggested, we have improved Fig. *2* by adding lower and upper bounds to the model predictions obtained by considering the whole range of the parameter uncertainties. The new figure shows how important it is to include the second-order terms, because neither model 1 nor model 2 can explain the experimental data. The robustness of this conclusion has improved with this sensitivity analysis.*

*A new Fig. *3* has been added to the manuscript following the advice of both referees. It shows three snapshots of the populations of virus and infected cells in space at different instant. This new figure provides a visualization of the expansion process, as well as a new way to compute the front speed for model 3.*

*Minor points have been take into account and corrected in the text.*

## Abbreviations

GBM: Glioblastoma
VSV: vesicular stomatitis virus
PDE: partial differential equation
PFU: plaque-forming unit
BHK: baby hamster kidney
HPI: hours post infection
GFP: green fluorescent protein
